# Understanding the Inflammatory Aspect of Osteoarthritis: Lessons from Immune Checkpoint Inhibitors

**DOI:** 10.3390/jcm15020658

**Published:** 2026-01-14

**Authors:** Daniel M. Portnoy, Matthieu Paiola, Carly Tymm, Robert Winchester, Adam Mor, Yevgeniya Gartshteyn

**Affiliations:** 1 Division of Rheumatology, Department of Medicine, Columbia University Irving Medical Center, New York, NY 10032, USA; 2 Herbert Irving Comprehensive Cancer Center, Columbia University Irving Medical Center, New York, NY 10032, USA

**Keywords:** osteoarthritis, immunotherapy, immune-related adverse events

## Abstract

Osteoarthritis (OA) is the most prevalent form of arthritis and is a major global health burden. OA is a heterogeneous condition with multiple contributing mechanisms that characterize different subtypes and stages of the disease. In this review, we examine the insights gained into the immunological characteristics of OA that have emerged from the increasingly widespread use of checkpoint inhibitors in the immunotherapy of malignancies. We discuss how the conventional view of OA as a degenerative disease is changing in view of the evidence suggesting that OA has an inflammatory component along with the presence in joint tissue of peripherally tolerized autoreactive resident memory T cells, which upon release of their inhibition by immunotherapy mediate immune-related adverse event arthritis (irAE-arthritis). We review clinical trials evaluating the efficacy of immunosuppressive therapies in modifying the course of OA, thereby providing an additional perspective on the presence and nature of the inflammation in OA. In summary, we argue that a shift from the traditional understanding of OA as a mechanical disease to one that incorporates the role of synovial immune cells and mechanisms of self-tolerance is necessary to guide future therapies, including the use of immune checkpoints for patients with OA.

## 1. Introduction

Osteoarthritis (OA) is the most common form of joint disease, affecting more than 10% of the population [1]. OA poses a significant burden for adults worldwide, leading to personal and socioeconomic challenges [2]. These include pain, fatigue, limited activity, reduced social participation, and a lower quality of life. Socioeconomic challenges encompass societal costs associated with both pharmacological and non-pharmacological treatments, which are further exacerbated by decreased productivity and employment among affected individuals. Although osteoarthritis is highly prevalent and linked to significant morbidity, its clinical heterogeneity continues to hinder a full understanding of its underlying mechanisms, leaving open the question of whether OA represents a single disease entity [3,4].

Osteoarthritis is characterized by the catabolic breakdown of joint tissue, associated with cartilage matrix degradation, with concurrent pathological remodeling leading to new bone formation that can be radiographically identified as osteophytes and subchondral sclerosis [5]. Current evidence shows that the prevalence of OA increases with age, obesity, and metabolic diseases, primarily affecting the knees, hips, spine, and the first carpometacarpal (CMC) and metatarsophalangeal (MTP) joints [1,6]. Other risk factors include a history of athletic or occupational stress, previous joint damage, genetic factors, and abnormal joint structure loading [7]. Indeed, there is a fivefold increased risk of developing OA in joints previously affected by injury; however, the mechanism behind this association remains unclear [8]. The primary objective of this review is to explore the latest advancements re-defining OA in the context of immunological dysregulation and to examine new immunomodulatory therapeutic options that directly target these immune-mediated pathways in OA. Importantly, we draw on insights from OA flares induced by immune checkpoint inhibitor (ICI) use in oncology to better understand how an ICI enhanced and prolonged immune response modifies the OA microenvironment and exposes contributing inflammatory mechanisms of this disease. By incorporating these new viewpoints, this review offers an updated perspective on OA as having an immunologic component with the potential for inducing inflammation when regulatory immune checkpoint pathways fail.

## 2. Immunology of Osteoarthritis: Evidence and Therapy

### 2.1. Traditional Understanding of Osteoarthritis

For years, OA was considered a predominantly a non-inflammatory condition resulting from mechanical of joint stress and cartilage damage [9]. Compared to inflammatory etiologies of joint pain, as seen in rheumatoid arthritis (RA) and psoriatic arthritis (PsA), OA is usually distinguished by the predominance of weight-bearing joint involvement, the absence of elevated inflammatory markers such as ESR and CRP, and the lack of constitutional symptoms that suggest systemic inflammation [10]. As a result, treatment of OA has focused on conservative measures of joint preservation and pain control. Interventions such as exercise, physical therapy, and weight loss serve as the first line of treatment, followed by non-steroidal anti-inflammatory drugs and other analgesics [11,12]. When conservative OA treatments are ineffective, surgical joint replacement is the only definitive therapy, highlighting the need for novel research-based interventions for OA [11]. Further research into the pathogenesis of joint destruction that underlies OA is crucial, as targeting novel pathways may lead to more effective treatments in the future.

### 2.2. The Association of Existing OA with the Development Inflammatory Arthritis Induced by the Use of Immune Checkpoint Inhibitors in Cancer Treatment Suggests That Immune Mechanisms Mediate OA Flares

The inspiration for this review evolved from our observation on the relationship between OA and the incidence of inflammatory arthritis following therapeutic use of immune checkpoint inhibitors in cancer treatment, thus raising a broader questions about the presence and nature of an inflammatory component of OA [13]. Immune checkpoint inhibitors (ICIs) are pharmacotherapeutic agents, most commonly monoclonal antibodies, that enhance anti-tumor T cell function by interfering with the inhibitory signaling pathways meant to down regulate the immune response following the initial activation. ICIs represent a groundbreaking advancement in cancer therapy, harnessing the body’s immune system to target and destroy cancer cells more effectively than traditional chemotherapy. They achieve this by blocking immune checkpoints that mediate (1) the inhibition of anti-tumor-targeted effector T cells and (2) the development of inhibitory regulatory T cells [14]. This is accomplished by inactivating the inhibitory T cell signaling pathways by targeting checkpoint molecules such as Programmed Cell Death Protein 1 (PD-1), Programmed Death-Ligand 1 (PD-L1), or Cytotoxic T-Lymphocyte Antigen 4 (CTLA-4). The subsequent increase in effector T cell activity unleashes a strong anti-tumor immune response.

During the generation of the T cell repertoire some T cells normally emerge from the thymus capable of recognizing intrinsic tissue antigens. These T cells become peripherally tolerized by the expression of PD-1 and take up residence in the tissue where they remain quiescent. However, following ICI administration these peripherally tolerized tissue resident T cells undergo activation and proliferation and may result in inflammation of non-tumor tissue that is clinically recognized as an immune-related adverse event (irAE). irAE-arthritis is one such example where there is evidence that the PD-1 expressing resident tissue cells drive the inflammatory response, as discussed in detail subsequently. irAE-arthritis, which can present as an oligoarticular or polyarticular arthritis similar to RA or psoriatic arthritis, can develop anytime following commencement of ICI therapy and often once initiated persists even after cessation of immunotherapy, suggesting lasting immunologic effect [14]. Importantly, development of irAE flares in only a subset of patients provides a rare opportunity to study immune-mediated mechanisms that predispose to inflammatory flares. Amongst patients with pre-existing rheumatologic disease, 1 in 3 develop a flare of their underlying autoimmune conditions following treatment with ICIs [15]. The development of irAE arthritis in the context of pre-existing OA thus provides a novel framework to explore the immune mechanisms that characterize and potentially bridge these two conditions.

Our group recently described a markedly increased prevalence of OA in patients who developed irAE-arthritis. The odds ratio for developing irAE-arthritis, compared to any other irAE, was 40.0 (95% CI 2.5–628.5, *p* = 0.009) for those with a history of OA in the involved joints versus those without [13]. Since the incidence of OA in the joint is not linked to an increased risk of cancer, this finding implies the association between OA and irAE related arthritis is specifically related to the initiation of ICI treatment, and presumably related to the release from inhibition of a tolerized self-reactive T cell [16]. A similar association between OA and ICI-induced joint pain was reported by Reid et al., coining the term “activated OA” (aOA). Specifically, aOA was suggested to be an independent irAE reflecting a worsening of OA in response to an increased number of patients developing OA pain after receiving checkpoint inhibitors [17]. We therefore believe that this work, along with our findings, links OA to the development of irAE-arthritis. The preferential development of inflammatory arthritis in OA-affected joints implies a molecularly distinct microenvironment poised for inflammatory activation and further supports the presence of an immunological mechanism implicated in the pathogenesis of OA.

Mechanistically, T cells harbor an inherent ability to activate in response to self-antigens because they enter the T cell repertoire by being positively selected on self-peptides during T cell development in the thymus. The potentially autoreactive T cells are subsequently either eliminated in the thymus by central tolerance or inactivated in the periphery at the tissue site where they encounter antigen. In the case of the OA joint, we envision that mechanical and oxidative stress leads to the post translational modification of matrix-derived molecules resulting in novel antigenic structures that can both stimulate the innate immune response and bind to the T cell receptors (TCR) of certain T cells that have not been tolerized to this novel self-antigenic structure. The resulting immune cell infiltration and cytokine production contribute to a pro-inflammatory micro-environment that undergoes peripheral tolerization. Peripheral T cell tolerance in the joint is mediated by the expression of inhibitory receptors, such as PD-1 on T cells, that dampens the immune response. The tolerized T cell becomes a PD-1 expressing tissue resident memory T cell (T_RM_) where its inflammatory activity remains checked. Administration of ICI therapy leads to the activation of these tolerized T cells residing in the already immune-activated OA microenvironment, leading to the breakdown of adaptive tolerance and irAE arthritis flare. In this context, the immune microenvironment in the OA joint becomes a hotbed for loss of self-tolerance and subsequent inflammatory flare. Below, we review the evidence supporting both innate immune system activation in OA, through synoviocytes, dendritic cell and macrophage dysregulation, as well as the evidence for an antigen driven adaptive immune response with clonal T-B cell proliferation and formation of secondary lymphoid structures in a subset of OA patients with more aggressive disease

### 2.3. A Complex Interplay of Innate and Adaptive Immune Dysregulation Characterizes Osteoarthritis

**Figure 1:**
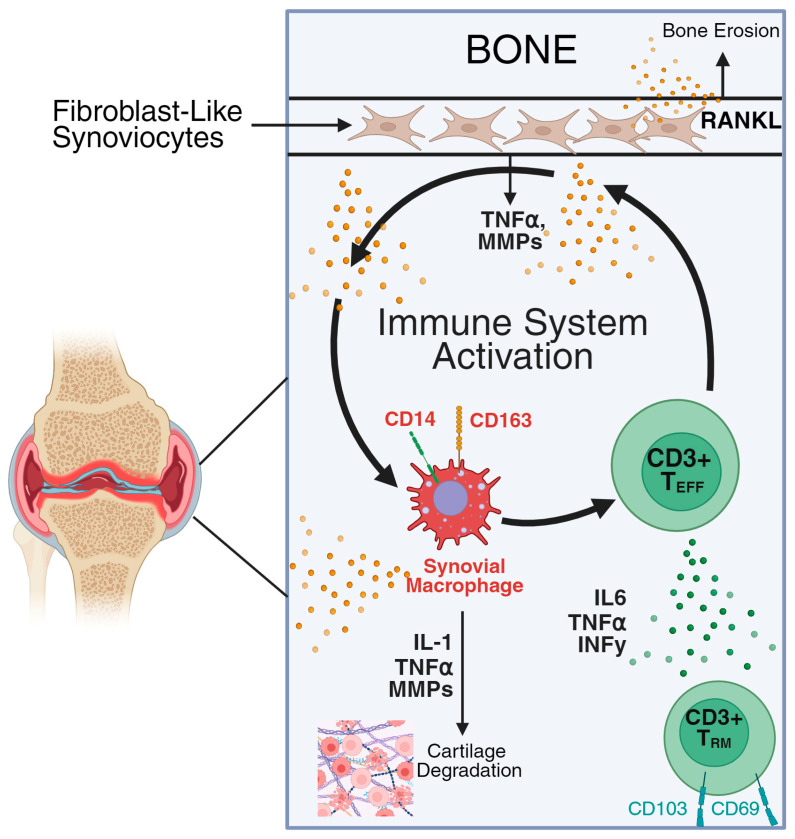
Immune Mechanisms in OA. The activation of synovial fibroblasts into an invasive, pro-inflammatory fibroblast-like synoviocytes (FLS) is marked by the synthesis of inflammatory cytokines such as TNFa that further amplify inflammation, metalloproteinases (MMPs) that degrade cartilage, and RANKL that drives bone resorption. Tissue-resident joint lymphoid and myeloid cells become expanded and activated, producing inflammatory cytokines that further drives immune cell infiltration and in situ proliferation. Tissue-resident memory T cells differentiate into pro-inflammatory effectors that synthesize TNFa and IFNy, support macrophage polarization and FLS activation, thereby perpetuating the cycle of inflammation and joint destruction.

Work over the last ten years has revealed the presence in OA of altered cell biologic and inflammatory characteristics [18,19] (Figure 1).

Reports identifying differentially methylated DNA isolated from human OA cartilage, as compared to healthy cartilage, revealed that an increased inflammatory process characterized a subset of patients with OA [20]. With the advent of next generation sequencing approaching, single-cell RNA sequencing analyses of cartilage and synovium have identified distinct clusters, or possibly stages, of OA joint pathology: some characterized by high inflammation as shown by enrichment of genes involved in pro-inflammatory pathways (cytokine–cytokine receptor interaction, chemokine signaling and osteoclast differentiation) and others by increased extracellular matrix remodeling and cell adhesion processes [21]. Damaged cartilage, as compared to healthy cartilage, has distinct cell-cell communication alterations enriched for pro-inflammatory and pro-degradative signaling [22,23]. Indeed, distinct cartilage cell populations may represent developmental stages of OA, from the pre-inflammatory and inflammatory chondrocytes that express IL-1 and TNFα receptors and may function to recruit immune cells, to the pre-hypertrophic and hypertrophic chondrocytes that produce matrix-degrading enzymes and drive pathological ossification [24]. Specifically, the underlying early inflammatory joint damage is associated with low-grade inflammation and enhanced macrophage activity [25,26]. Over time, local antigen presentation by the activated synovial macrophages and inflammatory fibroblast-like synoviocytes (FLS) leads to the engagement of the adaptive immune system, characterized by the presence of activated T cells in more aggressive OA disease [25,27,28,29]. The impact of this significant shift in understanding OA as an inflammation-mediated condition with distinct disease stages has yet to be fully incorporated into our clinical approaches for treating OA.

One of the inflammatory cellular changes that occurs in an OA joint is the activation of distinct subsets of synovial fibroblasts into an invasive, pro-inflammatory subset [30,31]. FLS are synovial fibroblasts that share some cell surface markers with fibroblasts, and which, under healthy conditions, synthesize matrix components such as hyaluronic acid, collagens, and fibronectin of the synovial fluid [30]. In the diseased joint, pathogenically activated and expanded FLS, engaged in cross-talk with synovial macrophages in situ, play a role in promoting the inflammatory and fibrotic response in OA [30,31]. Functional pathway analysis based on the transcriptional profiling of the OA joint revealed that fibroblast subsets isolated from painful OA sites promote fibrosis, inflammation, and the growth and activity of neurons [32]. Distinct populations of FLS in the joint have been described, corresponding to either a pro-inflammatory or a fibrotic phenotype [33,34]. The CD34^hi^ FLS, associated with the fibrotic group, appear to promote T-reg differentiation and attenuate inflammation [33]. Further differentiation can be made based on the expression of CD90, a cell surface glycoprotein also termed the THY1 antigen [31]. The CD34-THY1-FLS localize to the synovial lining layer (in contact with the intraarticular cavity), where they synthesize matrix components and contribute to joint homeostasis, but are also capable of producing metalloprotease and may thereby contribute to joint destruction [31]. On the other hand, the THY1^+^ FLS is a highly invasive and pro-inflammatory, cytokine-producing population that localizes to the sublining and is proposed to drive synovitis [35]. They are associated with pain, inflammation, and disease progression in OA [36]. In addition, these FLS secrete Dickkopf-1 (Dkk-1), a Wnt signaling pathway inhibitor which promotes angiogenesis but also increases cartilage degradation [35]. Dkk-1-producing fibroblasts are increased in OA synovial sublining and are capable of undergoing rapid expansion and conversion into myofibroblasts following mechanical stress, thereby driving inflammation and extracellular matrix remodeling [37].

In addition to FLS, OA joint inflammation may be mediated by infiltration and activation of immune cells in the joint. Analysis of healthy synovium has identified a population of tissue-resident lymphoid and myeloid cells that are readily isolated alongside the fibroblast and endothelial cells and become expanded with the development of OA [38]. In OA synovium, macrophages and T cells are the most abundant immune cells infiltrating the joint [39]. Activated macrophages have been for years recognized as key contributors to the pathogenesis of OA [40]. Using radiotracer imaging to detect activated macrophages, Kraus et al. provided in vivo evidence for the association between macrophage activity in the OA joint and pain severity [40]. During OA development, synovial macrophages become destabilized and activated through surface pattern recognition receptors (PRRs) that detect breakdown products from the degraded cartilage and extracellular matrix [25]. Upon activation, PRRs trigger downstream pathways, such as NF-κB, which drives the production of inflammatory cytokines, including TNFα and IL-1, thereby further contributing to in situ macrophage proliferation and polarization into an inflammatory subset [41,42]. Heterogeneous gene expression signatures of OA synovial macrophages can distinguish ‘inflammatory-like’ from ‘classical’ OA, suggesting distinct molecular macrophage activation mechanisms [43,44]. Specifically, opposing functions of MERTK^+^CD206^hi^ and MERTK^lo^CD206^lo^ macrophages in the joint, having tissue-resolving and proinflammatory characteristics, respectively, have been described [44]. The MERTK^lo^CD206^lo^ macrophages predominate in the inflammatory subtypes of OA and CD34^hi^ fibroblasts predominate in the fibrotic subtypes of OA [33].

Focusing on adaptive immunity, T and B cells play an intriguing and still incompletely defined role in the development and progression of OA [45,46]. An analysis of 185 end-stage OA synovial samples identified three histological classes, or pathotypes of OA: pauci-immune, diffuse-myeloid and lymphoid-myeloid [47]. The lympho-myeloid pathotype was associated with more aggressive and erosive disease, and more than half of the samples showed evidence of tertiary lymphoid structure formation. The majority of CD4+ T cells in the synovial fluid and synovial tissue have a memory phenotype, suggesting a developmental response to prior antigen recognition [48]. The majority of OA SF T cells highly express PD-1 suggesting a local chronic TCR mediated self-antigen-MHC-II complex recognition [13,49]. Indeed, clonal proliferation, defined by TRBV usage of activated T cells within the OA synovium, suggests presence of an immune response presumably directed against self-antigens in the joint [50]. Similarly, in another study, synovial infiltration of B lymphocytes was present in almost half of knee OA samples, with evidence of lymphoid follicles seen in the more severe cases [51]. Importantly, the analysis of CDR3 regions of rearranged VDJ genes from these B cells revealed oligoclonal B cell expansion suggesting that these were post-germinal center B cells that have been positive selected following antigen recognition and likely supported by cognate T-helper cells in situ. These findings support the presence of a sustained, antigen driven immune response that presumably contributes to disease progression in a subset of OA patients and likely is related to the peripheral tolerizing mechanisms of autoreactive T_RM_ discussed above.

### 2.4. Tissue-Resident Memory T Cells in the OA Synovium Provide a Mechanistic Link Between Pre-Existing OA and irAE-Arthritis

We analyzed the synovial fluid mononuclear cells (SFMCs) from OA patients and found the presence of tissue-resident memory T (T_RM_) cells, identified as CD103 and CD69 positive synovial T cells, that were also highly positive for PD-1 [13,52]. We also utilized a publicly available CITE-seq dataset of OA and RA synovial T cells to validate these synovial T_RM_ subsets, specifically PD-1, CD69, and CD103-expressing T cells within the OA joint [53]. In RA, the presence of these long-lived synovial T_RM_ cells persist in remission and mediate recurrent flares of inflammatory synovitis [54]. We thus infer that an OA joint similarly contains infiltrating T cells reactive against structural molecules in the joint to which they have insufficient self-tolerance. Thes structural antigens in the joint may arise developmentally or following stress-induced modification. The self-reactivity of these T cells results in their transient proliferation and maturation to an effector phenotype. At this point they are checked by the expression of inhibitory receptors such as PD-1, resulting in peripheral tolerance of these cells. They then remain in the joint tissues (synovium, entheses, associated adipose tissues) as CD103, CD69 and PD-1 expressing T_RMs_. Administration of ICI immunotherapy (i.e., anti-PD1) targets the expanded population of PD-1^+^ T cell subsets in the OA joint to proliferate and secrete pro-inflammatory cytokines, mediating inflammatory arthritis [13]. This is further supported by findings from other studies that have linked irAE-related colitis and dermatitis to the activation of in situ T_RM_ cells, implicating these cells in the mechanism of irAE-arthritis [55,56,57]. While additional research is necessary to gain a clearer insight into how T_RM_ cells develop within OA joint tissues and influence the onset of irAEs, the identification of PD1^+^ T_RM_ in OA, along with the observation that OA is a significant risk factor in irAE-arthritis, supports the classification of the OA joint as an immunological niche poised for activation, given an appropriate trigger [27].

The high expression of PD-1 in CD8 and CD4 T_RM_ cells in the tissue suggests that regulatory PD-1 signaling pathways function to maintain T_RM_ populations by fine tuning their self-reactive TCR signaling [49]. In fact, downstream PD-1 signaling pathways are required to maintain stem-like CD8 T cell memory both in cancer as well as in local tissues following resolution of acute infection [49,58,59]. The specific T_RM_ differentiation programs and phenotypes are heterogenous, tissue specific and mostly described for CD8 T cells. Upon entry into the tissue, IL-15, TGF-β or CD69 expression initiate tissue retention by downregulating S1PRs or by antagonizing their activity [60,61]. T_RM_ differentiation and maintenance further depends on the tissue expression of integrins CD103 or CD49a, as well as the upregulation of various transcription factors such as Blimp1, Hobit and Runx3 [61]. Interestingly, T_RM_ maintenance is also dependent on the antigen availability (i.e., TCR signal strength) [61]. In the arthritic joint, T_RM_ have been detected in RA, spondyloarthritis and psoriatic arthritis where their presence is presumed to mediate flare recurrence over time [54,62,63]. Nevertheless, their molecular requirements for differentiation and role in the human arthritic diseases remains to be clarified.

The identification of PD-1 expressing T_RM_ in the OA synovium suggests the presence of an immune microenvironment poised for activity in these injured joints. They are implicated with many autoimmune diseases such as psoriasis and Crohn disease [60,64,65]. Whether these T cells are secondarily responding to the same newly formed proinflammatory modified structures that first engage the innate immune system or whether the response is more primary in character reflecting a loss of adaptive tolerance is a currently an unanswered question. It is likely that the clusters of interacting T cells described decades ago by Sakkas and Platsoucas, which might now be designated as tertiary immune tissue, may be clusters derived from T_RM_ tolerized by the expression of PD-1 [28,66]. Indeed, the infiltration and/or local cross-differentiation from self-tolerant T_RM_ into pro-inflammatory CD4^+^ T cell helper subsets, secreting Th1, Th2, and Th17 cytokines, is seen in explanted OA joints and correlates with OA-related pain and disability [67]. These CD4^+^ T cells can trigger synovitis in the early stages of OA by secreting TNF-α, IFN-γ, and IL-17. The inflammatory cytokine network affects chondrocyte gene expression and reduces the production of proteoglycans, including aggrecan and type II collagen, which are crucial components of the extracellular matrix [68]. Th1 cells are particularly enriched in the OA synovium, where they secrete IFN-γ. After adjusting for T cell equivalents, one study demonstrated that IFNγ transcript levels in OA correspond to those in RA [28,66]. T cell-derived IL-17A contributes to the activation of the FLS, stimulating further release of pro-inflammatory developmental-polarizing cytokines [69]. Furthermore, IL-17 in the OA joint has been shown to induce pro-inflammatory chondrocyte development and enhance expression of catabolic factors, propagating destruction of cartilage in OA [70,71]. T cells, like macrophages, release chondro-destructive metalloproteinases into the OA synovium, thereby contributing to cartilage destruction in the joint [72]. Additionally, these activated T cells produce receptor activator of nuclear factor kappa-B ligand (RANKL), a TNF-family signaling molecule that activates osteoclasts, driving resorption of subchondral bone and joint destruction [73,74,75].

The presence and differentiation of CD8^+^ T cells, predominantly into IFNy and IL17 producing CD8^+^ T cells, is present in both the synovial membrane and synovial fluid of OA joints [76,77]. Cartilage degeneration is slower in a CD8^+^ T cell knockout mouse model of OA when compared to the control [78]. CD8^+^ T cells become activated in the inflammatory environment of the OA joint and are expanded during disease progression [78]. Still, fewer CD8^+^ T cells have been found in the OA joint and surrounding areas than CD4^+^ T cells [74,79].

Inflammation is implicated in the pathogenesis of OA, both within the joint microenvironment and, more broadly, on a systemic level where it may polarize lymphocyte development towards a more pathogenic phenotype. For example, patients with obesity, a condition known to promote inflammation, have a higher risk of developing OA of the hand. This joint is unlikely to endure mechanical stress from excess weight [18,80]. This phenomenon is believed to result from inflammation caused by adipokines like leptin, which is secreted by adipose tissue and is also increased in the synovium of OA patients, where the leptin level correlates with the grade of cartilage destruction [81]. Furthermore, in a mouse model with a genetic predisposition to Alzheimer’s, OA was shown to accelerate and amplify neuroinflammation, as evidenced by glial cell activation, quantification of inflammation-related mRNAs, and the size and number of amyloid plaques [82]. Finally, a prospective cohort study revealed that individuals who consume a diet with high inflammatory potential have an increased risk of developing new-onset knee OA, suggesting that engaging in activities that may cause inflammation is associated with the development of OA [83]. Overall, OA and inflammation appear to be intricately linked, manifesting on both macro and micro scales.

### 2.5. Using Immunosuppressive Therapies to Treat Osteoarthritis

Despite the growing evidence for an immune-mediated process driving OA progression, significant molecular heterogeneity of this disease has made it challenging to identify a uniform mechanism for therapeutic targeting. Analysis of OA tissue has identified molecularly different patient clusters that suggest heterogeneity between patients and/or between the early vs. late stages of the disease [21]. While immune system involvement predominates in some patients, dysregulated pathways of cartilage degradation, extracellular matrix remodeling, bone resorption and repair/fibrosis contribute in different extents across patient subgroups [47]. There is also heterogeneity in the immune cell populations that predominate in the synovial tissue across different biopsies [84]. Sampling the OA joint at different stages of the diseases adds additional complexity given lineage plasticity of joint chondrocytes, fibroblasts and infiltrating immune cells over time [85,86]. The anatomical site, history of excess load or traumatic injury to the joint, gender and presence of underlying inflammatory features are additional sources of clinical complexity. The determination of these clinical and molecular characteristics and their function in predicting clinical response to anti-inflammatory therapies, although currently overlooked in most clinical trials to date, will likely shape the future of precision medicine in OA.

One of the most general and potent anti-inflammatory agents are corticosteroids, which function to inhibit immune cell activation and function. According to the American College of Rheumatology guidelines, intra-articular (IA) glucocorticoid injections for treating hip and knee OA are strongly recommended [87]. Multiple systematic reviews agree with this conclusion, however emphasizing that pain relief is achieved in only a subset of patients and at best provides only short-term relief [88,89,90]. Interestingly, IA corticosteroids can also be effective for treating OA in canines [91]. The exact mechanism of intraarticular corticosteroid efficacy remains unknown.

Attempts at using disease-modifying antirheumatic drugs (DMARDs) as a treatment for OA produced often inconclusive and less impressive results than they do in inflammatory types of arthritis such as rheumatoid or psoriatic arthritis [92]. One DMARD that was initially suggested for the treatment of OA is hydroxychloroquine (HCQ). HCQ was considered a potential treatment for OA due to its inhibitory action on Toll-like receptor (TLR) signaling, which regulates macrophage activation in OA [93]. However, despite its initial promise, HCQ was found to have no benefit in reducing pain, nor was it effective in improving physical function or quality of life [93,94,95].

Alternatively, reports of effective immunosuppressive medication use in treating osteoarthritis have emerged. We conducted a clinicaltrials.gov search to identify clinical trials in osteoarthritis involving the use of immunosuppressive therapies, which we have summarized below [Table 1].

The use of methotrexate in treating knee and hand OA produced mixed results [96,97,98,112]. In one multicenter, randomized-controlled study, the addition of oral MTX (titrated up to 25 mg once a week) to the standard of care for knee OA induced a significant improvement in pain and function after six months [97]. More recently, methotrexate failed to improve pain or synovitis in knee OA—however its important to note that over 40% of the patients in this study had advanced OA possibly reflecting end-stage disease with complete cartilage loss [96]. In another randomized, placebo-controlled trial from Australia, oral MTX at 20 mg once a week for hand OA was effective in reducing pain and stiffness [98]. Conversely, MTX at a lower dose of 10 mg once a week did not reduce pain or risk of progressive joint erosions in patients with erosive hand osteoarthritis [99]. Overall, although several trials have shown that using MTX for knee OA and hand OA can have therapeutic benefits, we still lack the clinical biomarkers to precisely identify the subset of patients whose disease is driven by immune cell dysregulation and thus most likely to respond to cell-based DMARD therapy in OA.

Biological agents, such as anti-TNFα therapy, represent a mechanistically driven therapeutic alternative in the treatment of OA, given the above data. TNFα, secreted by FLS, macrophages, and T cells in OA, triggers the production of nitric oxide (NO), cyclooxygenase-2 (COX-2), and prostaglandin E2 (PGE2) synthesis, and a variety of cytokines, all of which contribute to tissue inflammation and cartilage destruction [113]. Efficacy of TNFα inhibitors in OA, however, has shown conflicting results. A double-masked, randomized clinical trial found that etanercept dosed 50 mg subcutaneously weekly for 24 weeks was not effective in relieving pain for patients with erosive hand OA [100]. In contrast, another study evaluating a single intra-articular etanercept injection directly into the knee joint in moderate to severe knee OA found that patients experienced significantly greater pain relief after the etanercept injection than an HA injection [101]. Intraarticular injection of infliximab, administered directly into the hand joints in erosive hand OA, was also effective in reducing pain in a small pilot study. In this study, one arm was randomized to receive infliximab, and the other received saline injections [105]. Adalimumab, a monoclonal anti-TNFα antibody, has also been studied in OA with mixed results. In OA of the hand, subcutaneous administration of adalimumab was not found to have any benefit compared to placebo, while in moderate to severe OA of the knee, intra-articular administration of adalimumab was found to have better clinical outcomes than HA in improving pain and function [102,103,104]. In a network meta-analysis on the efficacy of anti-inflammatory biologic agents for OA, researchers analyzed 1566 patients from 15 randomized controlled trials to find that etanercept and infliximab, both anti-TNFα targeted therapies, were superior to placebo for knee OA pain [114]. This suggests that a background activation of TNFα-producing macrophages and Th1 T cells can mediate pain in OA joints and that neutralization of inflammatory cytokines, particularly with intra-articular monoclonal antibodies, can be an effective treatment strategy for a subset of patients.

While many therapeutics appear promising in the development of OA treatments, others have been deemed unsuccessful in clinical trials. For example, the activation of the NLRP3 inflammasome in the OA joint has made IL-1α a treatment target [115]. However, intra-articular injection of anakinra, an IL-1 receptor agonist, tested at 50 mg and 150 mg doses in knee OA was not significantly different from placebo [106]. Additionally, the bispecific antibody lutikizumab, which targets IL-1α and IL-1β, was also found to be ineffective for OA, as it did not improve pain, synovitis, or function in the knee or hand [107,108,116]. Another cytokine present in the OA joint and identified as a possible therapeutic target in OA is IL-6 [117]. However, a recent randomized, placebo-controlled study found that tocilizumab, an anti-IL-6 monoclonal antibody dosed intravenously as an 8 mg/kg monthly infusion, was ineffective for pain relief in hand OA [109].

Yet another unmet need in OA therapy is the reduction in subchondral bone resorption that results in a particularly aggressive subtype of OA known as “erosive” osteoarthritis. In a recent randomized, double-blind, placebo-controlled phase IIa trial for erosive hand OA, denosumab, a RANKL inhibitor, was found to reduce radiographic erosive progression compared to placebo [110]. Additionally, extended use of the drug also led to significant improvements in both pain and disability levels. By inhibiting RANKL, osteoclast maturation, function, and survival are affected, thereby reducing the rate of bone resorption [118]. While denosumab is traditionally prescribed for osteoporosis to mitigate fracture risk, the promising results from this study may broaden its indication for use in OA.

The efficacy of glucagon-like peptide-1 (GLP1)-agonists for pain relief in OA has also been reported [119]. In a recent Phase 3 clinical trial that enrolled patients with obesity and knee osteoarthritis, treatment with the once-weekly GLP-1 agonist semaglutide resulted in more significant reductions in body weight and pain related to knee OA compared to placebo [111]. In addition to their well-established role in weight loss, GLP-1 agonists have immunomodulatory effects. GLP-1 agonists bind at the GLP-1 receptor where they increase intracellular cyclic AMP levels and inhibits downstream NFKB and MAPK signaling pathways resulting in the decreased production of pro-inflammatory cytokines to reduce inflammation [120]. GLP-1R is expressed on articular chondrocytes, and signaling is associated with preventing apoptosis, anti-inflammatory activity, and matrix protection [121].

## 3. Conclusions and Future Directions

Osteoarthritis is a complex heterogeneous disease with a multifaceted pathology. Once considered noninflammatory, OA is now recognized as having an inflammatory component characterized by synovitis, inflammatory FLS and macrophage subsets, cytokine production, and the presence of T cells [18,84]. Evidence is increasing that inflammation appears to have a role in cartilage destruction and subsequent joint space narrowing. Furthermore, the identification of PD-1^+^ T_RM_ cells along with pro-inflammatory macrophages in the OA joint suggests the presence of an immune microenvironment within the OA synovium, with peripherally tolerized T cells poised to react to pro-inflammatory stimuli. In this context, our identification of OA as a risk factor for irAE-arthritis in cancer patients treated with ICIs is particularly relevant in underscoring the inflammatory potential within the T_RM_ of the OA joint [13]. With the newly recognized role of immune system activation in the pathogenesis of OA, novel directions for future work arise. It remains unknown whether inflammatory and non-inflammatory OA are distinct conditions or rather opposite extremes on a continuum of disease progression. Similarly, the transitions between molecularly distinct inflammatory subsets of OA development over time are not completely understood. Furthermore, while the inflammatory phenotypes of OA have been linked with more aggressive clinical disease, it is still not known whether immunosuppressive therapy can slow down joint destruction and alter the progression of OA. Translating the molecular and cellular findings into actionable clinical biomarkers has been particularly challenging given the heterogeneity between patients, joints and OA stages. These same challenges have also limited our ability to infer events occurring specifically within joint tissue at any single biopsy time point. Despite this, synovial biopsies can provide potential biomarkers to guide therapy in the future, such as specific cytokine signatures or expanded pathogenic cell subsets from the joint. The possibility that a small subset of circulating T cells with distinctive TCR sequences will serve as a biomarker of the immune population in the joint is raised by the identification of some unique clonotype in the blood of patients before ICI therapy, which then expand greatly during the ensuing irAE arthritis [122]. Importantly, we lack non-invasive methods to stratify OA disease pathotypes, further limiting the ability to deliver precision-based therapeutic interventions in the absence of biopsy information. For patients receiving ICI therapy, the benefit of screening for advanced OA prior to ICI use is unclear given lack of preventive strategies. Nevertheless, with the evolving understanding of the immune mechanisms that drive OA progression, the possibility of a precision-based treatment approach becomes a feasible goal for the future.

OA has an extensive impact on systemic health, linking it to osteoporosis, sarcopenia, cardiovascular diseases, diabetes mellitus, neurological disorders, and mental health conditions [123]. Recognizing shared inflammatory mechanisms and reclassifying OA as an inflammatory disease or a disease with inflammatory features would broaden our understanding of its pathogenesis and support improved research, treatment, and long-term outcomes. Given that OA is one of the leading causes of disability worldwide, effectively combating this disease requires proper public education, starting with dispelling the “wear-and-tear” epithet and recognizing it as a disease with a latent inflammatory component.

## Figures and Tables

**Table 1:** Immunosuppressive Therapies in OA.

Study	Intervention	Mechanism	Indication	Dosage	Frequency Interval	Duration Used	Route	N	Outcome	Limitations
Zhu et al. [96], 2025	MTX	Anti-metabolite	Knee	15 mg	Once weekly	12 months	Oral	215	Compared to placebo, oral MTX use did not reduce pain scores or radiographic effusion/synovitis findings on MRI.	More than 40% of the enrolled patients had severe OA (defined by the Kellgren-Lawrence score of 4), possibly indicating end-stage disease.
Kingsbury et al. [97], 2024	MTX	Anti-metabolite	Knee	6-week escalation 10 mg to 25 mg	Once weekly	12 months	Oral	155	Oral MTX added to usual medications demonstrated statistically significant reduction in knee OA pain, stiffness, and function at 6 months.	Benefits of MTX at 6 months did not sustain at 12 months; trial allowed patients to remain on their pre-existing analgesic regimen, which introduces potential confounders for MTX effect.
Wang et al. [98], 2023	MTX	Anti-metabolite	Hand	20 mg	Once weekly	6 months	Oral	97	Treatment of hand osteoarthritis and synovitis with 20 mg MTX for 6 months had a moderate but potentially clinically meaningful effect on reducing pain.	Study was initially designed to last 2 years, but it was cut short by the COVID-19 pandemic; swollen joint counts could not be assessed virtually during lockdowns as well.
Ferrero et al. [99], 2021	MTX	Anti-metabolite	Hand	10 mg	Once weekly	12 months	Oral	64	No statistically significant differences were observed between the MTX and placebo groups in terms of function.	The dose of methotrexate was low and potentially subtherapeutic.
Kloppenburg et al. [100], 2018	Etanercept	Anti-TNF	Hand	50 mg for 24 weeks, then 25 mg	Once weekly	1 year	SQ	90	Anti-TNF did not relieve pain effectively after 24 weeks.	Lowering the dose to 25 mg/wk may have reduced the efficacy of the drug.
Ohtori et al. [101], 2015	Etanercept	Anti-TNF	Knee	10 mg	Once	4 weeks	SQ	39	Direct injection of etanercept into OA knee joints was an effective treatment for pain in moderate and severe OA patients.	Small number of patients recruited. Outcome assessment was at 1 month after the injection with no long-term follow-up.
Chevalier et al. [102], 2015	Adalimumab	Anti-TNF	Hand	40 mg	2 injections at a 15-day interval	6 months	SQ	78	Adalimumab was not superior to placebo in alleviating pain in patients with hand OA not responding to analgesics and NSAIDs.	Two injections may not be sufficient to invoke any clinical benefits.
Aitken et al. [103], 2018	Adalimumab	Anti-TNF	Hand	40 mg	Biweekly	12 weeks	SQ	43	Adalimumab did not show any effect on pain, synovitis or bone marrow lesions in patients with erosive hand OA with MRI-detected synovitis as compared to placebo after 12 weeks.	Baseline synovitis was moderate, so inflammation may have been too low to detect meaningful change; X-rays were not scored for disease severity; small sample size.
Wang [104], 2018	Adalimumab	Anti-TNF	Knee	10 mg	Once	4 weeks	IA	56	Adalimumab by intra-articular injection was effective and tolerated for moderate to severe knee osteoarthritis.	Relatively small sample size; adalimumab and hyaluronic acid were only administered once at baseline; influence of adalimumab treatment dose was not evaluated.
Fioravanti et al. [105], 2009	Infliximab	Anti-TNF	Hand	0.2 mL (0.1 mg/mL)	Monthly	12 months	IA	10	At 6 months all the patients experienced relief from spontaneous pain and pain on lateral pressure in the hand treated with infliximab and these findings became statistically significant after 1 year.	Very small sample size
Chevalier et al. [106], 2009	Anakinra	IL-1 Receptor Agonist	Knee	50 mg or 150 mg	Once	12 weeks	IA	170	Anakinra was well tolerated as a single 50-mg or 150-mg intraarticular injection in patients with OA of the knee. However, anakinra was not associated with improvements in OA symptoms compared with placebo.	Anakinra has a short half-life, so the potential benefit may have been missed at later timepoints; potential benefit 4 days after administration was seen.
Fleischmann et al. [107], 2019	Lutikizumab	Anti-IL-1α & IL-1β	Knee	25, 100, & 200 mg	Biweekly	50 weeks	SQ	350	The WOMAC pain score at week 16 had improved significantly versus placebo with lutikizumab 100 mg but not with the 25 mg or 200 mg doses. Beyond week 16, the WOMAC pain score was reduced in all groups but was not significantly different between lutikizumab-treated and placebo-treated patients.	Discrepancy between ultrasound and MRI grading of synovitis; approximately two-thirds of patients had relatively mild radiographic changes and none had severe disease, thus limiting the likelihood of lutikizuumab demonstrating significant improvements in pain.
Kloppenburg et al. [108], 2019	Lutikizumab	Anti-IL-1α & IL-1β	Hand	200 mg	Biweekly	24 weeks	SQ	132	Despite adequate blockade of IL-1, lutikizumab did not improve pain or imaging outcomes in erosive hand OA compared with placebo.	More time may have been needed to observe a radiographic effect of lutikizumab.
Richette et al. [109], 2021	Tocilizumab	Anti-IL-6	Hand	8 mg/kg	2 infusions 4 weeks apart	12 weeks	IV	91	Tocilizumab was no more effective than placebo for pain relief in patients with hand osteoarthritis.	Study design was underpowered to detect smaller effect sizes; 2 infusions may be insufficient to inhibit IL-6R.
Wittoek et al. [110], 2024	Denosumab	Anti-RANKL	Hand	60 mg	Every 3 months	48 weeks	SQ	100	Denosumab reduced radiographic erosive progression in erosive hand OA versus placebo without increased toxicity. Also, fewer erosive joints developed through week 48 in the denosumab group and led to significant improvement in pain and disability levels in the extension phase through week 96	Single center study; needs to be replicated.
Bliddal et al. [111], 2024	Semaglutide	GLP-1 Agonist	Knee	2.4 mg	Once weekly	68 weeks	SQ	407	Among participants with obesity and knee osteoarthritis with moderate-to-severe pain, treatment with once-weekly injectable semaglutide resulted in significantly greater reductions in body weight and pain related to knee osteoarthritis than placebo.	Lack of imaging and assessment of metabolic and inflammatory markers at follow-up, so the effect of semaglutide on OA pathophysiology could not be determined; changes in outcome not assessed after the end of the treatment period.

N, number of patients enrolled; MTX, methotrexate; TNF, Tumor Necrosis Factor; GLP-1, Glucagon-Like Peptide 1; SQ, subcutaneous injection. IA, intra-articular injection. IV, intravenous infusion.

## Data Availability

No new data were created or analyzed in this study.
